# Using Natural Deep Eutectic Systems as Alternative Media for Ocular Applications

**DOI:** 10.3390/pharmaceutics15051553

**Published:** 2023-05-21

**Authors:** Célia Sarmento, Hugo Monteiro, Alexandre Paiva, Ana Rita C. Duarte, Ana Rita Jesus

**Affiliations:** LAQV-REQUIMTE, Chemistry Department, NOVA—School of Science and Technology, 2829-516 Caparica, Portugal; cc.sarmento@campus.fct.unl.pt (C.S.); h.monteiro@campus.fct.unl.pt (H.M.); abp08838@fct.unl.pt (A.P.); ard08968@fct.unl.pt (A.R.C.D.)

**Keywords:** natural deep eutectic systems, ocular formulations, alternative solvents, biocompatible, acetazolamide, drug delivery

## Abstract

The major goal of this work was to study the potential of natural deep eutectic systems (NADES) as new media for ocular formulations. In formulating eye drops, it is important to increase the retention time of the drug on the surface of eye; hence, due to their high viscosity, NADES may be interesting candidates for formulation. Different systems composed of combinations of sugars, polyols, amino acids, and choline derivatives were prepared and then characterized in terms of rheological and physicochemical properties. Our results showed that 5–10% (*w*/*v*) aqueous solutions of NADES have a good profile in terms of viscosity (0.8 to 1.2 mPa.s), osmolarity (412 to 1883 mOsmol), and pH (7.4) for their incorporation of ocular drops. Additionally, contact angle and refractive index were determined. Acetazolamide (ACZ), a highly insoluble drug used to treat glaucoma, was used as proof-of-concept. Herein, we show that NADES can increase the solubility of ACZ in aqueous solutions by at least up to 3 times, making it useful for the formulation of ACZ into ocular drops and thereby enabling more efficient treatment. The cytotoxicity assays demonstrated that NADES are biocompatible up to 5% (*w*/*v*) in aqueous media, promoting cell viability (above 80%) when compared to the control after 24 h incubation in ARPE-19 cells. Furthermore, when ACZ is dissolved in aqueous solutions of NADES, the cytotoxicity is not affected in this range of concentrations. Although further studies are necessary to design an optimal formulation incorporating NADES, this study shows that these eutectics can be powerful tools in the formulation of ocular drugs.

## 1. Introduction

More than 2 billion people in the world suffer from some type of vision impairment or blindness, and at least half of them have a preventable condition [1]. Cataracts are the most prevalent condition, followed by glaucoma, diabetic retinopathy, and age-related macular degeneration (ARMD) [2]. Vision impairment affects people of all ages, although the majority of those affected are over 50.

Most age-related eye diseases cannot be reversed, but in some cases vision can be partially restored using proper treatment. In several circumstances frequent injections are required, but the risk of infection after the treatment is significant and, consequently, patients must be carefully monitored. Others can only be treated through surgery [3]. Nevertheless, for the treatment of some conditions, such as glaucoma, topical eye drops remain the most convenient route for ocular drug administration, especially considering patient compliance; however, the physiology of the eye limits their use. When a drop of drug formulation is administered, the majority of the drug is lost to precorneal drainage and only a small portion is absorbed at the nasolacrimal duct, where it becomes systemically available [4]. This is why the design of new drug delivery systems for ocular applications remains an active research field and why the development of new strategies for the management of ocular diseases, with the ultimate goal of improving the quality of life of patients, is still needed.

Herein, we propose the use of natural deep eutectic systems (NADES) as new media for ocular formulations, especially for ocular drops, to overcome their lack of efficiency and improve the solubility and bioavailability of therapeutic compounds. 

Ocular formulations usually contain viscosity enhancers in their composition [5], and NADES have an intrinsic viscosity that can be tailored by the addition of water, avoiding the incorporation of unnecessary additives or significantly decreasing their concentration, leading to enhanced final formulations.

NADES are a new class of alternative solvents, composed of two of more compounds that, once mixed at specific molar ratios, suffer a significant depression of their melting point, becoming liquid at room temperature. They are mainly composed of natural metabolites such as sugars, amino acids, and polyols, and have emerged as alternative solvents for many applications, especially in the pharmaceutical field, mostly because they are considered non-toxic, biocompatible, and biodegradable [6,7,8,9,10,11,12]. NADES can be easily designed for a specific task, and this is one of their major advantages. Furthermore, their production is cost-effective since there is no waste produced and they do not require any purification steps; thus, they can be considered more sustainable materials [13]. Additionally, NADES are already known as solubilizing agents for biomolecules and insoluble pharmaceuticals [14,15,16,17,18]. 

In this work, we studied the physical and chemical properties of several NADES and their aqueous solutions, including their viscosity, osmolarity, and contact angle, among others. We then discussed the differences between the systems and compared the results with easily accessed ocular drops, such as Tobrex^®^.

As proof-of-concept, we propose the use of NADES as solubility enhancers for acetazolamide (ACZ), an old-age treatment for glaucoma. ACZ is commonly administrated in tablet form, mainly to its low solubility, and presents a variety of side effects. We studied the solubility of ACZ in pure NADES and then the ACZ/NADES mixtures in aqueous media. We have also studied the toxicity of these mixtures and the pure systems in human retinal cells (ARPE-19). 

## 2. Materials and Methods

### 2.1. Materials

Betaine anhydrous (≥99%, Sigma, St. Louis, MO, USA), trehalose dihydrate (Hashibara, Japan), Glycerol (99.5%, Scharlau, Barcelona, Spain), Ethylene Glycol (≥99.5%, Carlo Erba, Emmendingen, Germany), D-(+)-glucose anhydrous (≥97.5%, Farma-Química, Guatemala), D-(-)Fructose (Sigma), D(+)-Sucrose (99.5%, Sigma), L-Proline (99%, Alfa Aesar, Ward Hill, MA, USA), *N*-Acetylcysteine, (Sigma), D-Sorbitol (98%, Sigma-Aldrich), HPMC (hydroxypropyl methylcellulose, Sigma), acetazolamide (≥99%, Sigma). A commercial sample of Tobrex^®^ (Novartis, Basel, Switzerland) was acquired and used as a control. 

### 2.2. Methods

#### 2.2.1. Preparation of NADES

The different NADES were prepared according to the procedure of previous studies [13]. Different compounds were combined at specific molar ratios by slowly mixing and heating the mixture to between 35 and 60 °C, with constant stirring, until a clear liquid was obtained. The water content of the NADES was then determined using an 831 KF Coulometer (Metrohm) with a generator electrode and without a diaphragm (Table 1).

#### 2.2.2. Preparation of NADES Solutions

To prepare the formulations, NADES were diluted in an artificial tears solution (AT)–0.9% NaCl (PanReac AppliChem)—at 5 and 10 % (*w*/*w*) concentrations to perform viscosity and osmolality analyses.

To study the pH, density, surface tension, contact angle, and refractive index, NADES were diluted in phosphate-buffered saline (PBS, Sigma) at 5 and 10% (*w*/*v*) concentrations.

#### 2.2.3. Rheological Studies

The rheological studies of the different systems in aqueous solutions were carried out using an Anton Paar Modular Compact Rheometer 102 fitted with parallel plate geometry with a 49.954 mm diameter (PP50, Anton Paar) and 0.5 mm gap. Before each measurement, samples were trimmed and then stabilized.

The viscosity vs. temperature measurements were performed after 5 min of stabilization at 15 °C under a constant shear rate of 10 s^−1^. Temperatures were raised at a rate of 1.1 °C/min from 15 to 40 °C. The viscosity vs. shear rate measurements were performed after 5 min of stabilization at 0 s^−1^ at a constant temperature of 25 °C. The shear rate was raised from 0 to 100 s^−1^ at a rate of 4.35 s^−1^/min. 

The same study was performed with a 0.3% (*w*/*v*) HPMC solution in PBS and with Tobrex^®^, which were used for comparison. All data is represented as the average of three measurements.

#### 2.2.4. Osmolarity, pH, Density, Surface Tension, Contact Angle, and Refractive Index 

Osmolarity analysis of the aqueous solutions of NADES was performed using a KNAUER Freezing Point Osmometer K-7400S. Osmometer measurements range from 0 to 2000 mOsmol/kg with a resolution of 1 mOsmol/kg. The calibration curve was performed using water (Carlo Erba Reagents, HPLC plus) as the 0 mOsmol/kg solution in addition to supplied standard solutions (400 and 850 mOsmol/kg). The values presented are an average of three measurements.

The pH measurements of the aqueous formulations were performed using a Metrohm 914 pH/Conductometer. All measurements were performed at room temperature (22–25 °C). The values presented are an average of three measurements. 

The density measurements of aqueous solutions of NADES were performed with an Anton Paar Stabinger Viscometer 3001 from 20 to 60 °C at a temperature rate of 10 °C/point. Each value results from an average of three measurements.

The surface tension measurements were performed using a standalone force tensiometer (Biolin Scientific Sigma 702) with a Du Noüy ring using the Huh–Mason correction. The values given are an average resulting from three consecutive measurements at 25 °C (controlled by a RW-0525G Lab Companion). 

The contact angle acquisition was performed using an optical goniometer (CAM 100, KSV Instruments Ltd.) that captured the drop image, while the respective software, CAM 100, calculated the value of the contact angle based on the width and height of the drop. For each drop, 10 frames were captured with an interval of 1000 ms between them. The method for contact angle measurement followed a sessile drop procedure on a polytetrafluoroethylene (PTFE) surface. The contact angle values are an average of three measurements. 

The refractive index measurements of the aqueous solutions of NADES were performed at room temperature using a monochromatic Abbe-2WAJ Refractometer. Triplicates were measured.

#### 2.2.5. Solubility Studies of ACZ in NADES

Acetazolamide was dissolved in different NADES at concentrations of 10 mg/g, 20 mg/g, and 30 mg/g. The mixture was heated up to 40 °C to facilitate the dissolution, due to the NADES high viscosity. Then, 5% (*w*/*v*) aqueous solutions of ACZ/NADES mixtures were prepared. Samples of these solutions were centrifuged at 12,000 rpm for 10 min and then diluted (1:10) in methanol and quantified using UV-Vis spectroscopy (288 nm). A calibration curve of ACZ was previously prepared in concentrations ranging from 12.5 to 150 µg/mL, ensuring the linearity of the method. A 5 mg/mL stock solution of ACZ in DMSO was prepared. Dilutions were prepared in MeOH. 

#### 2.2.6. In Vitro Cytotoxicity Evaluation

The cytotoxicity effects of NADES and ACZ/NADES mixtures were evaluated using the ARPE-19 cell line (ATCC), which is an immortalized human spontaneously arising retinal pigment epithelia cell line.

ARPE-19 cells were incubated for 24 h in a 96-well plate at a density of 1.0 × 10^4^ cells/well and then incubated for another 48 h until confluency was reached (ca. 70%). Afterwards, selected NADES and NADES/ACZ mixtures were added at 5% (*w*/*v*) and 10% (*w*/*v*) concentrations and incubated for 24 h at 37 °C and 5% CO_2_. Samples were prepared by dissolving the samples in culture media. 

Control cells were incubated with complete media. To evaluate cell viability, CellTiter 96^®^ AQ_ueous_ One Solution Cell Proliferation Assay (Promega), based on MTS (3-(4,5-dimethylthiazol-2-yl)-5-(3-carboxymethoxyphenyl)-2-(4-sulfophenyl)-2H-tetra-zolium), was carried out. 

#### 2.2.7. Statistical Analysis

The statistical analysis was carried out using GraphPad Prism 8.0.1 (GraphPad Software). The data presented is expressed as mean ± standard deviation (SD). Statistically significant differences were always evaluated against the control sample. It was considered statistically significant when *p*-values < 0.05. To perform this test a two-way ANOVA following the appropriate multiple comparison test was employed. Statistically significant differences are represented by asterisks.

## 3. Results and Discussion

In this study, several NADES were prepared, and the components of these systems were chosen based on our previous works [11,19,20] regarding their biocompatibility. Further, betaine was specifically chosen as a component because it has been shown to act as an osmoprotector in primary human corneal epithelial cells (HCECs) [21]. 

To select the working concentrations of NADES, the literature data was revised, especially in terms of cytotoxicity. In a previous study from our research group, Jesus et al. [12] studied the cytotoxic effects of different sugar-based NADES in L929 cells, a cell line that is frequently employed for biocompatibility studies, and it was shown that cells can withstand high concentrations of NADES without affecting their viability. NADES at 5% concentrations did not affect the cell viability, while in some cases using 10% (*w*/*v*) concentrations caused a slightly increased toxicity. Considering this, all the studies were carried out using the NADES presented in Table 1, at 5% and 10% (*w*/*v*) concentrations.

Although it has been reported that there is a disruption of the hydrogen bond network when a NADES is dissolved in water, Pereira et al. [22] showed that the eutectic system is not completely disrupted and that the system retains its properties. 

### 3.1. Rheological Studies

Viscosity is a crucial property of topical eye drops, as high viscosity corelates to longer precorneal residence time. However, high viscosities might cause pain, discomfort, and eye injury. The goal is to find a reasonable gap between an improved retention time and the occurrence of adverse effects.

Shear rate is an essential factor to consider when working with rheology and is defined as the velocity gradient. Ocular surface shear conditions range from 0.03–0.14 s^−1^ at rest, when the eye is immobile, to significant shears imposed while blinking, 4000 and 28,000 s^−1^ [9]. Human tear viscosity was studied throughout several shear rates [8,9]. In general, all tear samples exhibited significant shear-thinning, with viscosity declining from around 5 mPa.s at the lowest shear rate to about 1.5 mPa.s at the maximum shear rate. Ocular drops should, hence, have a high viscosity at a low shear rate, approaching that of natural tears, which helps to reduce blurring and significant discomfort when blinking [23]. Compounds with these characteristics should present shear-thinning behavior, being non-Newtonian fluids. 

Some authors studied the rheological properties of NADES and observed that, at high shear rates, the flow behavior of the tested NADES systems is temperature and shear rate independent [24,25,26,27]. 

**Figure 1:**
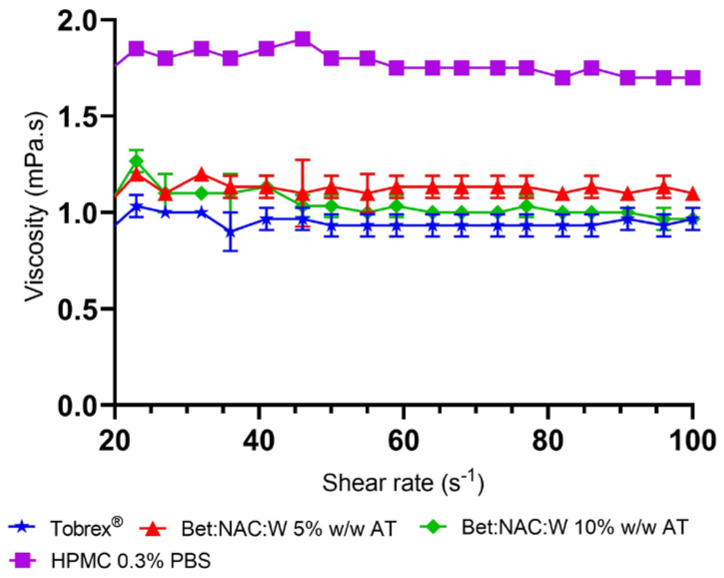
Viscosity of Bet:NAC aqueous solutions as a function of shear rate at 25 °C in comparison with Tobrex^®^ and HPMC (0.3% *w*/*v*). Data indicated as mean ± SD.

Nonetheless, in our work, a slight shear-thinning effect was observed, mainly at shear rates below 1 s^−1^. The commercial sample, Tobrex^®^ Ophthalmic Solution (Tobramycin 0.3%, Novartis), was used as a comparison. In Figure 1 it is possible to see an example of these observations. The remaining studies are presented in the Supplementary Information (S1 and Table S1).

The viscosity values at a shear rate above 20 s^−1^ were similar to or slightly higher than Tobrex^®^’s and in the same range of values as human tears, whereas, at the higher shear rates, NADES viscosity ranged between 0.8 mPa.s and 1.2 mPa.s. 

The same trend was observed for the other samples. At the highest shear rate measured, all NADES solutions had viscosities between 0.8 and 1.1 mPa.s. The highest viscosity NADES formulations are Bet:NAC 5% (*w*/*w*) AT, Glc:Pro:Gly 10% (*w*/*w*) AT, and Gly:Glc 10% (*w*/*w*) AT, all at 1.1 mPa.s. Nevertheless, the differences between our samples and Tobrex^®^ are almost insignificant. 

**Figure 2:**
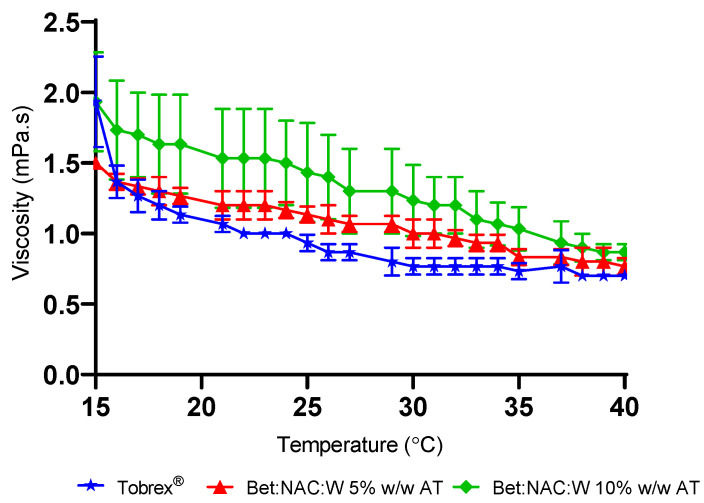
Viscosity of Bet:NAC aqueous solutions vs. temperature at 10 s^−1^ in comparison with Tobrex^®^. Data indicated as mean ± SD.

However, to better understand how different a formulation is, in terms of viscosity, we have also compared our NADES solutions to HPMC solution, which is one of the most common viscosity enhancers in ocular drops. Thus, we have studied the viscosity of HPMC at 0.3 % (*w*/*v*) concentration, which is the concentration normally reported in commercial samples. Figure 2 shows that HPMC viscosity also displays very slight shear-thinning behavior although it presents higher viscosity values than the Bet:NAC solution. 

Additionally, it is important to know how the viscosity of NADES is affected by the temperature, which influences the bioavailability of the active ingredient. As an example, in Figure 2 it is shown that the viscosity profile of Bet:NAC at 5% and 10% (*w*/*v*) concentrations is slightly higher than Tobrex^®^. 

Moreover, the viscosity data from the remaining systems are presented in Figure S2 (Supplementary Information), and it is possible to observe that the Gly:Glc 10% (*w*/*w*) AT system has the highest viscosity at 15 °C, while the Bet:NAC 10% (*w*/*w*) AT system has the highest viscosity at 40 °C. Moreover, at 5% (*w*/*w*) concentration, Bet:Suc:Gly has the highest viscosity of all systems measured. Nevertheless, all systems showed slightly higher viscosities than Tobrex^®^, especially at higher temperatures. This range of temperatures was selected considering the potential for different values during winter and summer and the average physiological temperature. 

### 3.2. Osmolality, pH, and Refractive Index

The eye surface is in a constant dynamic of production, evaporation, absorption, and drainage of tears. This can be translated by one single property, the final product of tear dynamics: osmolality [28]. The systems from Table 2 were all measured by freezing point osmometry and all NADES formulations presented osmolalities higher than the commercial sample Tobrex^®^; hence, they can be considered hyperosmolar when compared to human tears, which have an osmolality of 304 mOsmol/kg [29]. 

At both 5% and 10% (*w*/*v*) concentrations, the system that presented the lowest osmolality was the Fru:Glc:Suc system. Generally, systems with glycerol had increased osmolality values; however, the Bet:Gly 5% (*w*/*w*) AT system was an exception. 

Nevertheless, a study from Dutescu et al. [30] showed that there are hypo- and hyperosmolar ocular drops available on the market. In fact, in this study, the authors compared 87 commercial samples and concluded that the sample with the lowest osmolality had an osmolality of 131 mOsmol/L, while the highest osmolality was 1955 mOsmol/L, showing an enormous disparity between the samples. In vitro studies suggest that hypertonic drops can modify tear osmolality and consequently cause inflammation; however, the rapid clearance makes prediction of the in vivo effects difficult. The authors concluded that cytotoxicity is far more important than the osmolarity of a formulation. This study suggests that these high values of osmolarity are not a limitation of using NADES in a future formulation. 

Furthermore, our samples did not significantly change the pH of a phosphate-buffered solution, showing that they do not have an impact on physiological pH levels. The only exception was for the Bet:NAC system, which had a substantial effect, lowering the pH to 2.6 (Table 2). *N*-acetyl-cysteine (NAC) is used as a medicine to treat paracetamol overdose and also has notable antioxidant and anti-inflammatory capabilities [31]. Despite decreasing the pH, Bet:NAC it did not show signs of cytotoxicity towards ARPE-19 cells, as shown in Section 3.5. Additionally, in ocular formulations, excipients such as hydrochloric acid and sodium hydroxide are commonly used to adjust the final formulation pH. Therefore, the value obtained for this specific NADES is not a concern. 

Another relevant property of an ocular formulation is the refractive index. The action of light refraction is what makes the functionality of the whole eye possible. The tear film acts as the first refractive component of the eye, and changes in its dynamics might result in both visual and ocular surface discomfort [32]. Therefore, the refractive index of NADES solutions was measured and is presented in Table 2. The values determined for the refractive index did not differ significantly within the systems tested. Overall, these values are slightly higher than that of tear film, which is reported as 1.337 [29], and quite similar to that of water, which is 1.3326 [33]. It can also be observed that the refractive index increases with the increase in NADES concentration. Patel et al. [34] reviewed a large volume of data, concluding that the refractive index of the tear film’s aqueous layer gradually increases from the underside of the lipid layer in the direction of the cornea; specifically, the precorneal tear film has a refractive index of about 1.482 above a layer where the average refractive index is about 1.337. Having NADES results in this range suggests that these systems would not cause blurring and discomfort when used as delivery systems. 

### 3.3. Density, Surface Tension, and Contact Angle

**Figure 3:**
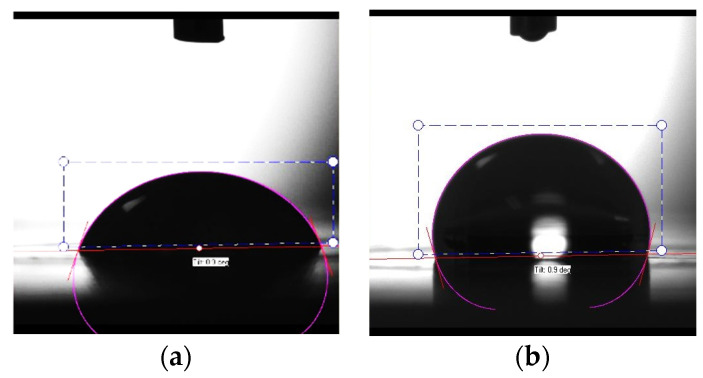
Contact angle of (**a**) Tobrex^®^ and (**b**) Bet:NAC at 5% (*w*/*v*) concentration, measured on a PTFE surface at room temperature.

To develop ocular drops that properly adhere to the cornea, both the cornea surface and the tear film must be considered and kept stable and consistent over time. Instillation of a formulation can disrupt the tear film over time and lead to cell damage that consequently causes conditions such as dry eye disease [35]. Surface tension allows us to understand the stability of the tear film and the tear film break-up time, which influences the eye drops’ spreading ability and adherence [36]. The contact angle is directly related to wettability, which is the ability of a liquid to spread over a surface. The study of the systems designed is presented in Table 3. Figure 3 presents the contact angle of (a) Tobrex^®^ and (b) 5% (*w*/*v*) aqueous solution of the Bet:NAC system as an example.

Regarding surface tension, lower values lead to quick spreading over the ocular surface without blinking. The physiological range at the air/tear fluid interface is 40–46 mN/m [36], which is considerably lower than the results obtained for the systems tested herein, which were closer to water values (ca. 72 mN/m at room temperature) [37]. Surface tension above the physiological range is anticipated to result in the appearance of tear film instability, which is correlated with dry eye syndrome [36]. Additionally, higher values of surface tension led to an increase in the drop volume instillation. A superior drop volume increases the amount of drug released in a single dose. Furthermore, a higher drop volume increases the washout, which has the opposite effect: loss of drug intake [38]. Thus, this property must be brought closer to physiological values without compromising the other parameters. It is important both to have a stable tear film and to maintain the efficacy of the eye drops, which can be easily compromised. Han et al. [39] evaluated several ophthalmic formulations available on the market. Interestingly, despite the expectation that the surface tension range would be very similar to normal tears, the reality was different, with many formulations showing higher surface tension values. For example, TheraTears^®^ Lubricant Eye Drops presented a surface tension of 70.9 mN/m, even higher than most of our NADES solutions. In light of this, our NADES solutions presented values of surface tension within the range of values found in commercial samples; therefore, surface tension values do not pose a limitation for NADES application in ocular formulations. However, in a future formulation it will be possible to slightly decrease the surface tension through the addition of a small percentage of a polymer or another substance. Concerning the contact angle, some experimental considerations must be made before analyzing the results. The fact that contact angles could only be measured using the sessile drop method may lead to inconsistencies with the in vivo reality. It would be ideal to use the captive bubble method, which allows the surface to be hydrated, making it thus more analogous to in vivo conditions; however, while applying this method, it is also necessary to consider the surface used. 

Although the aim of having good wettability is to achieve contact angles lower than 90°, this requires testing on a surface that is generally comparable to reality, which did not occur in this case. The results presented here show prominent values on the hydrophobic PTFE surface, resulting in an opposite effect (Figure 3). In this case, it can be concluded that the systems’ formulations have poor wettability on hydrophobic surfaces, which, for the intended application, is a good result. This means that NADES formulations present hydrophilic properties, which are necessary for their application as ophthalmic solutions. Bock et al. [35] used the sessile drop method to test four commercial eye drops on three different surfaces. A hydrophobic polyethylene terephthalate, such as PTFE, was used, as well as glass, which is hydrophilic, and cell monolayers of human corneal epithelial cells. All samples presented similar values, and the biggest change was the surfaces used to acquire these contact angle values. The contact angles on the glass surface ranged from 42.9° to 48.9°, while those on the hydrophobic surface ranged from 105.7° to 112.1°, coincident with the values that were obtained in this work for a similar surface. The same authors observed that when eye drops reached the monolayer of corneal cells, they spread throughout the surface immediately. Consequently, it can be postulated that these NADES formulations may behave in the same manner under diverse conditions. HPMC measurements also sustain that this polymer has properties better suited to ophthalmic formulations. It would be necessary to determine the behavior of NADES combined with polymers, not only to enhance viscosities but also to promote better surface tension for uniform dispersion of water-insoluble particles in aqueous NADES solutions [40]. 

### 3.4. Acetazolamide Solubility Studies

Acetazolamide (ACZ) is considered insoluble in water since its water solubility is reported as lower than 1 mg/mL. For this reason, ACZ is orally administrated to treat acute glaucoma; however, some studies showed 80–100% of patients reported side effects, such as paraesthesias (burning or prickling sensation that is usually felt in the hands, arms, legs, or feet), dysgeusia (taste disorder), polyuria (frequent urination), and fatigue [41]. Therefore, the topical application of ACZ is desirable.

**Figure 4:**
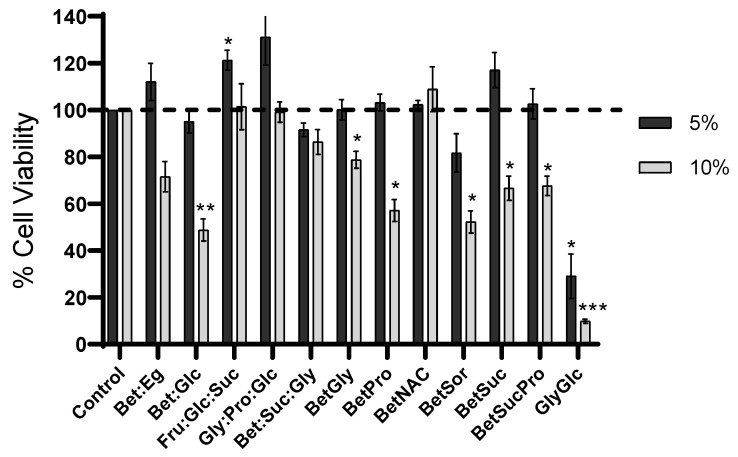
Cell viability of ARPE-19 cells after 24 h of incubation with different concentrations of NADES. Data represents mean ± SD (n = 3). Statistically significant differences were determined by Dunnet’s multiple comparisons test and are represented by asterisks: * *p* < 0.05; ** *p* < 0.01; *** *p* < 0.001, two-way ANOVA. The absence of asterisks means that there are no significant differences when compared with control values at both concentrations. Dotted line is the control line, i.e., the 100% of cell viability.

In this work we have dissolved ACZ in the systems shown in Table 1, at levels of 10, 20, and 30 mg ACZ/g of NADES (Figure 4). The solubility determination was based on visual assessment. Then, 5% (*w*/*v*) aqueous solutions of ACZ/NADES mixtures were prepared for the systems in which ACZ was completely soluble. At this point, considering all the results presented so far, we decided to study the solubility of ACZ only in 5% (*w*/*v*) aqueous solutions. The quantification of ACZ in aqueous solutions (Table S2, Supplementary Information) was carried out spectroscopically at λ_max_ = 288 nm using a calibration curve previously prepared (Supplementary Information Figure S4 and Table S2). 

Table 4 summarizes the qualitative solubility results for ACZ in pure NADES as well as in the aqueous solutions. 

For reference, the solubility of ACZ in water was also quantified by dissolving 10 mg/mL ACZ in water. Then, the same procedure was followed for the quantification of ACZ in water. Using this method, ACZ had a water solubility of 0.7 mg/mL, which is quite similar to the reported value (0.72 mg/mL) [42]. 

The quantitative solubility of ACZ in aqueous solutions of ACZ/NADES mixtures is presented in Table S2 (Supplementary Information). The results show that, using NADES, it was possible to increase the solubility of ACZ in aqueous media by at least up to 3 times, representing a major advantage of using NADES in ocular formulations. 

Interestingly, some of the systems that showed improved physico-chemical properties, such as Fru:Glc:Suc, did not show improvement in their solubility. Additionally, some systems were also removed from the study due to cytotoxicity, as shown in the next section, including the Bet:Sor and Gly:Glc systems. The Bet:Eg, Bet:Glc, Bet:NAC, and Bet:Gly systems were the most promising ones in terms of improving the solubility of ACZ.

### 3.5. Cytotoxicity Evaluation of NADES and ACZ/NADES Mixtures

The cytotoxic profile of NADES was determined in ARPE-19 cells following the MTS assay. Overall, the systems showed no significant cytotoxicity at 5% (*w*/*v*) concentration except for the Gly:Glc system, which presented higher toxicity in ARPE-19 cells, a different outcome from the one reported by Jesus et al. [12]. Additionally, the Bet:Sorb system also affected the cell viability, with values around 80% (Figure 4). In the study using NADES at 10% (*w*/*v*) concentrations, the Fru:Glc:Suc, Glc:Pro:Gly, Bet:Suc:Gly, and Bet:NAC systems presented cell viabilities around or above 100%. The remaining systems presented significantly lower cell viability values.

**Figure 5:**
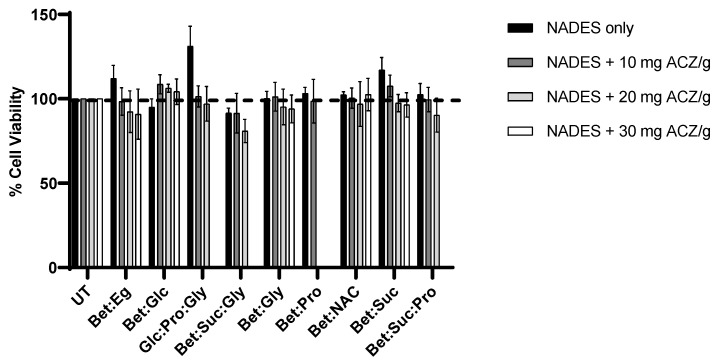
Cell viability of ARPE-19 cells after 24 h of incubation with 5% (*w*/*v*) concentration NADES/ACZ mixtures. Data represents mean ± SD (n = 3). Statistically significant differences were determined by Tukey’s multiple comparisons test, two-way ANOVA. The absence of asterisks means that there are no significant differences when compared with control values at all concentrations. Doted line referred to the control i.e., the 100% of cell viability.

Regarding the cytotoxicity of ACZ/NADES mixtures, we have only tested the 5% aqueous mixtures with high solubility for ACZ, i.e., only the samples marked with the “++++” symbol in Table 4 were studied. Figure 5 shows the cell viability values for these mixtures. 

From these results, we can conclude that the addition of ACZ to NADES does not significantly increase the cytotoxicity toward retinal cells, since most of the mixtures present cell viabilities >90%. 

## 4. Conclusions

The results presented in this work suggest that NADES are potential excipients for ocular formulations, especially ocular drops. NADES solutions have low cytotoxicity, especially at 5% (*w*/*v*) concentration. It was observed that these presented Newtonian behaviour, yet at the concentrations tested, the viscosity values are lower than expected but still within the range found in commercial samples of eye drops. The fact that NADES solutions are hyperosmolar is a certain concern; however, there are commercially available ocular formulations with higher osmolarities, suggesting that this characteristic will not be a limitation to their use. Regarding the pH and refractive index, we have shown that these are within the optimal range; additionally, the higher values of surface tension presented should not be a limitation, and, using appropriate additives, it would be possible to decrease these values to optimal ones. Overall, all systems presented a similar behaviour in terms of physico-chemical properties. 

However, when these systems are used to formulate a drug, such as acetazolamide (ACZ), which is highly insoluble in aqueous media, some differences are observed between them. For example, the Glc:Pro:Gly system showed the best results in terms osmolality and cytotoxicity at low concentrations, but it showed less stability over time and low ability to improve ACZ solubility. Nevertheless, several NADES improved the solubility of ACZ by at least up to 3 times in water. 

In summary, considering all the results together, the most promising systems for the envisaged application are the Bet:NAC and Bet:Gly systems. Although further studies are still necessary to improve the residence time of drugs on the ocular surface as well as to guarantee the safety of these systems for ocular tissues, this study opens new opportunities for the use of NADES in ocular applications. 

Furthermore, the major advantages of using NADES as excipients for ocular drops are that they improve the solubility of hydrophobic drugs and confer a certain viscosity to the system that may decrease the amount of additives needed. Additionally, NADES are easily prepared, biocompatible, eco-friendly, and cheap alternative solvents.

Researchers are still searching for alternative formulations for ocular applications with increased drug residence times and increased bioavailability, aiming at the development of more efficient and patient compliant therapies that will diminish the need for invasive procedures. We believe that NADES can contribute to those developments and help to address the many problems encountered during the formulation of ocular drugs. 

## Figures and Tables

**Table 1:** Summary of the NADES prepared and their water content (%).

NADES	Components			Molar Ratio	Water Content (%)
	A	B	C		
Bet:Eg	Betaine	Ethylene glycol	-	1:3	0.80 ± 0.1
Bet:Glc	Betaine	Glucose	-	5:2	16.4 ± 0.1
Fru:Glc:Suc	Frutose	Glucose	Sucrose	1:1:1	19.4 ± 2.4
Glc:Pro:Gly	Glucose	Proline	Glycerol	3:5:3	20.1 ± 0.6
Bet:Suc:Gly	Betaine	Sucrose	Glycerol	2:1:3	10.0 ± 0.1
Bet:Gly	Betaine	Glycerol	-	1:2	1.70 ± 0.1
Bet:Pro	Betaine	Proline	-	1:2	12.9 ± 2.9
Bet:NAC	Betaine	*N*-acetyl cysteine	-	1:1	17.7 ± 1.1
Bet:Sorb	Betaine	Sorbitol	-	1:1	13.8 ± 1.5
Bet:Suc	Betaine	Sucrose	-	4:1	16.7 ± 0.4
Bet:Suc:Pro	Betaine	Sucrose	Proline	5:2:2	18.5 ± 0.3
Gly:Glc	Glycerol	Glucose	-	4:1	0.20 ± 1.0

**Table 2:** Osmolality, pH, and refractive index values. NADES samples in AT. Data indicated as mean + SD.

Sample	Osmolality [mOsmKg^−1^]		pH		Refractive Index	
Tobrex^®^	214 ± 1		7.47		1.3379	
Artificial Tears (AT)	286 ± 1		7.42		1.3350	
HPMC 0.3% in PBS	280 ± 1		-		1.3359	
**NADES**	**5% *w*/*w* AT**	**10% *w*/*w* AT**	**5% *w*/*w* AT**	**10% *w*/*w* AT**	**5% *w*/*w* AT**	**10% *w*/*w* AT**
Bet:Eg	962 ± 3	1883 ± 1	7.43	7.52	1.3402	1.3466
Bet:Glc	602 ± 4	947 ± 3	7.32	7.29	1.3459	1.3463
Fru:Glc:Suc	412 ± 2	558 ± 2	7.32	7.29	1.3459	1.3463
Glc:Pro:Gly	538 ± 2	825 ± 2	7.28	7.20	1.3416	1.3470
Bet:Suc:Gly	639 ± 2	1032 ± 4	7.37	7.37	1.3397	1.3491
Bet:Gly	432 ± 4	1504 ± 4	7.46	7.42	1.3416	1.372
Bet:Pro	587 ± 2	957 ± 1	7.38	7.33	1.3433	1.3460
Bet:NAC	563 ± 2	900 ± 4	2.65	2.64	1.3407	1.3455
Bet:Sorb	585 ± 1	922 ± 7	7.43	7.43	1.3405	1.3460
Bet:Suc	554 ± 2	875 ± 3	7.46	7.45	1.3411	1.3456
Bet:Suc:Pro	541 ± 5	833 ± 2	7.42	7.42	1.3405	1.3460
Gly:Glc	709 ± 3	1207 ± 8	7.38	7.28	1.3411	1.3456

**Table 3:** Density, surface tension, and contact angle values of NADES solutions. Data indicated as mean + SD.

Sample		Density (g/cm^3^)	Surface Tension (mN/m)	Contact Angle (θ)
Tobrex^®^		n.a.	n.a.	74.7 ± 5.0
HPMC 0.3% in PBS		n.a.	43.82 ± 0.02	57.7 ± 1.4
Bet:Eg	5% *w*/*w* in PBS 10% *w*/*w* in PBS	1.0118 1.0172	68.18 ± 0.03 69.49 ± 0.01	105.3 ± 1.9 101.7 ± 0.8
Bet:Glc	5% *w*/*w* in PBS 10% *w*/*w* in PBS	1.0150 1.0209	71.57 ± 0.01 70.01 ± 0.08	105.9 ± 1.7 106.9 ± 1.2
Fru:Glc:Suc	5% *w*/*w* in PBS 10% *w*/*w* in PBS	1.0192 1.0328	70.71 ± 0.01 70.68 ± 0.08	104.3 ± 1.3 103.0 ± 3.1
Glc:Pro:Gly	5% *w*/*w* in PBS 10% *w*/*w* in PBS	1.0171 1.0287	63.28 ± 0.02 60.28 ± 0.05	104.2 ± 0.2 100.7 ± 3.0
Bet:Suc:Gly	5% *w*/*w* in PBS 10% *w*/*w* in PBS	1.0165 1.0277	70.31 ± 0.04 68.96 ± 0.09	102.2 ± 0.9 104.4 ± 0.1
Bet:Gly	5% *w*/*w* in PBS 10% *w*/*w* in PBS	1.0148 1.0239	66.23 ± 0.02 66.68 ± 0.07	106.3 ± 2.0 107.3 ± 0.2
Bet:Pro	5% *w*/*w* in PBS 10% *w*/*w* in PBS	1.0129 1.0197	69.24 ± 0.03 68.13 ± 0.09	109.9 ± 0.1 108.0 ± 3.4
Bet:NAC	5% *w*/*w* in PBS 10% *w*/*w* in PBS	1.0148 1.0230	68.51 ± 0.03 67.09 ± 0.05	105.7 ± 3.0 103.0 ± 3.1
Bet:Sorb	5% *w*/*w* in PBS 10% *w*/*w* in PBS	1.0167 1.0221	68.44 ± 0.07 66.30 ± 0.08	108.8 ± 2.0 103.2 ± 3.0
Bet:Suc	5% *w*/*w* in PBS 10% *w*/*w* in PBS	1.0150 1.0246	70.94 ± 0.02 69.10 ± 0.04	103.3 ± 2.5 104.9 ± 1.6
Bet:Suc:Pro	5% *w*/*w* in PBS 10% *w*/*w* in PBS	1.0162 1.0258	66.30 ± 0.05 66.51 ± 0.04	101.9 ± 2.3 108.0 ± 1.3
Gly:Glc	5% *w*/*w* in PBS 10% *w*/*w* in PBS	1.0189 1.0316	70.70 ± 0.02 64.17 ± 0.06	103.0 ± 2.9 103.9 ± 1.1

**Table 4:** Qualitative solubility of acetazolamide in pure NADES and in 5% aqueous solutions of NADES.

NADES	Solubility in Pure NADES			Solubility in 5% Aqueous Solutions		
	10 mg/g	20 mg/g	30 mg/g	10 mg/g (0.5 mg ACZ/mL)	20 mg/g (1 mg ACZ/mL)	30 mg/g (1.5 mg ACZ/mL)
Bet:Eg	++++	++++	++++	++++	++++	++++
Bet:Glc	++++	++++	++++	++++	++++	++++
Fru:Glc:Suc	n.s.	n.s.	n.s	n.a.	n.a.	n.a.
Glc:Pro:Gly	++++	++++	n.s.	++++	++++	n.a.
Bet:Suc:Gly	++++	++++	n.s.	++++	++++	n.a.
Bet:Gly	++++	++++	++++	++++	++++	++++
Bet:Pro	n.s.	n.s.	n.s.	n.a.	n.a.	n.a.
Bet:NAC	++++	++++	++++	++++	++++	++++
Bet:Sorb	n.s.	n.s.	n.s.	n.a.	n.a.	n.a.
Bet:Suc	++++	++++	++++	++++	++++	++
Bet:Suc:Pro	++++	++++	n.s.	++++	++++	n.a.
Gly:Glc	++++	n.s.	n.s.	++++	n.a.	n.a.

++++ highly soluble (no particles visible); ++ sparingly soluble (some particles in suspension); n.s.—not soluble; n.a.—not analized.

## Data Availability

Data are available in a publicly accessible repository.

## Supplementary Materials

The following supporting information can be downloaded at: https://www.mdpi.com/article/10.3390/pharmaceutics15051553/s1, Figure S1: Viscosity as function of shear rate of NADES solutions and Tobrex^®^; Figure S2: Viscosity vs. temperature of NADES solutions and Tobrex^®^; Figure S3: Example of ACZ dissolved in NADES; Figure S4: Calibration curve of Acetalozamide; Table S1: Experimental viscosity data of Tobrex^®^, HPMC and NADES solutions (5% and 10%, *w/v*); Table S2: Quantification of ACZ in 5% aqueous solutions of NADES/ACZ mixtures
