# Supplementary material for: Using Natural Deep Eutectic Systems as Alternative Media for Ocular Applications
Source: Pharmaceutics. 2023 May 21;15(5):1553. doi: 10.3390/pharmaceutics15051553 (PMC10223266; doi:10.3390/pharmaceutics15051553)
Supplement: Supplementary file 1 [file pharmaceutics-15-01553-s001.zip › pharmaceutics-2359689-supplementary.pdf]

Using Natural Deep Eutectic Systems as Alternative Media for Ocular Applications

Célia Sarmento, Hugo Monteiro, Alexandre Paiva, Ana Rita C. Duarte and Ana Rita Jesus *

LAQV-REQUIMTE, Chemistry Department, NOVA—School of Science and Technology, 2829-516 Caparica, Portugal; cc.sarmento@campus.fct.unl.pt (C.S.); h.monteiro@campus.fct.unl.pt (H.M.); abp08838@fct.unl.pt (A.P.); ard08968@fct.unl.pt (A.R.C.D.)

* Correspondence: ar.gameiro@fct.unl.pt; Tel.: +351-212-948-564

Supporting Information

S1 Viscosity as a function of shear rate

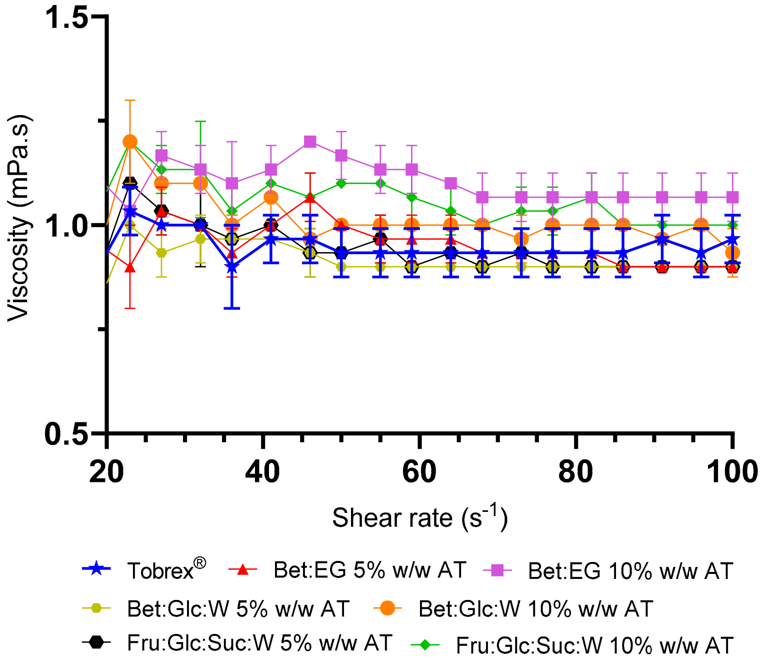
1.5
1.0
0.5
Viscosity (mPa.s)
20
40
60
80
100
Shear rate (s-1)
Tobrex®
Bet:EG 5% w/w AT
Bet:EG 10% w/w AT
Bet:Glc:W 5% w/w AT
Bet:Glc:W 10% w/w AT
Fru:Glc:Suc:W 5% w/w AT
Fru:Glc:Suc:W 10% w/w AT

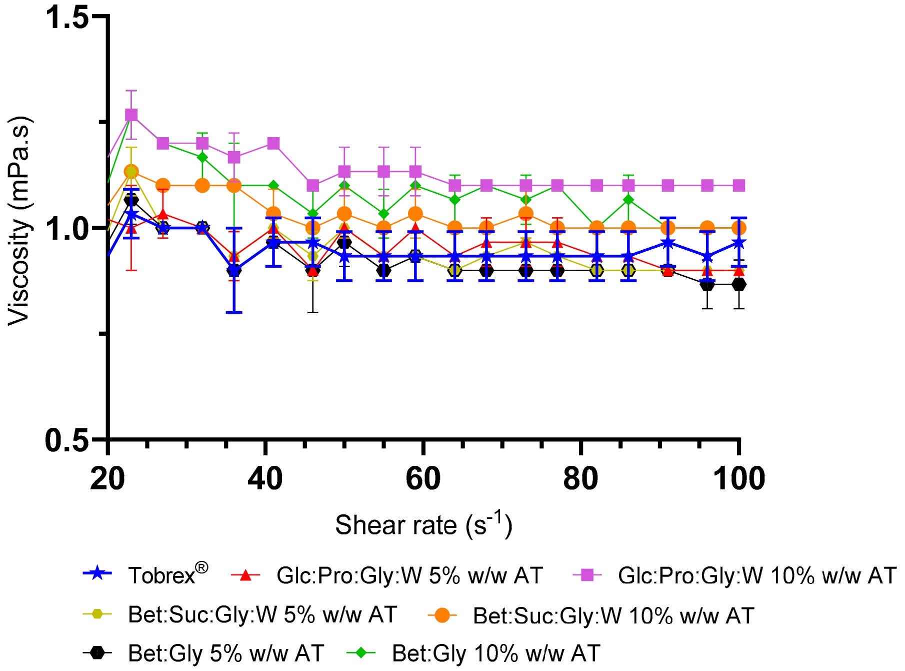
1.5
1.0
0.5
Viscosity (mPa.s)
20
40
60
80
100
Shear rate (s-1)
Tobrex®
Glc:Pro:Gly:W 5% w/w AT
Glc:Pro:Gly:W 10% w/w AT
Bet:Suc:Gly:W 5% w/w AT
Bet:Suc:Gly:W 10% w/w AT
Bet:Gly 5% w/w AT
Bet:Gly 10% w/w AT

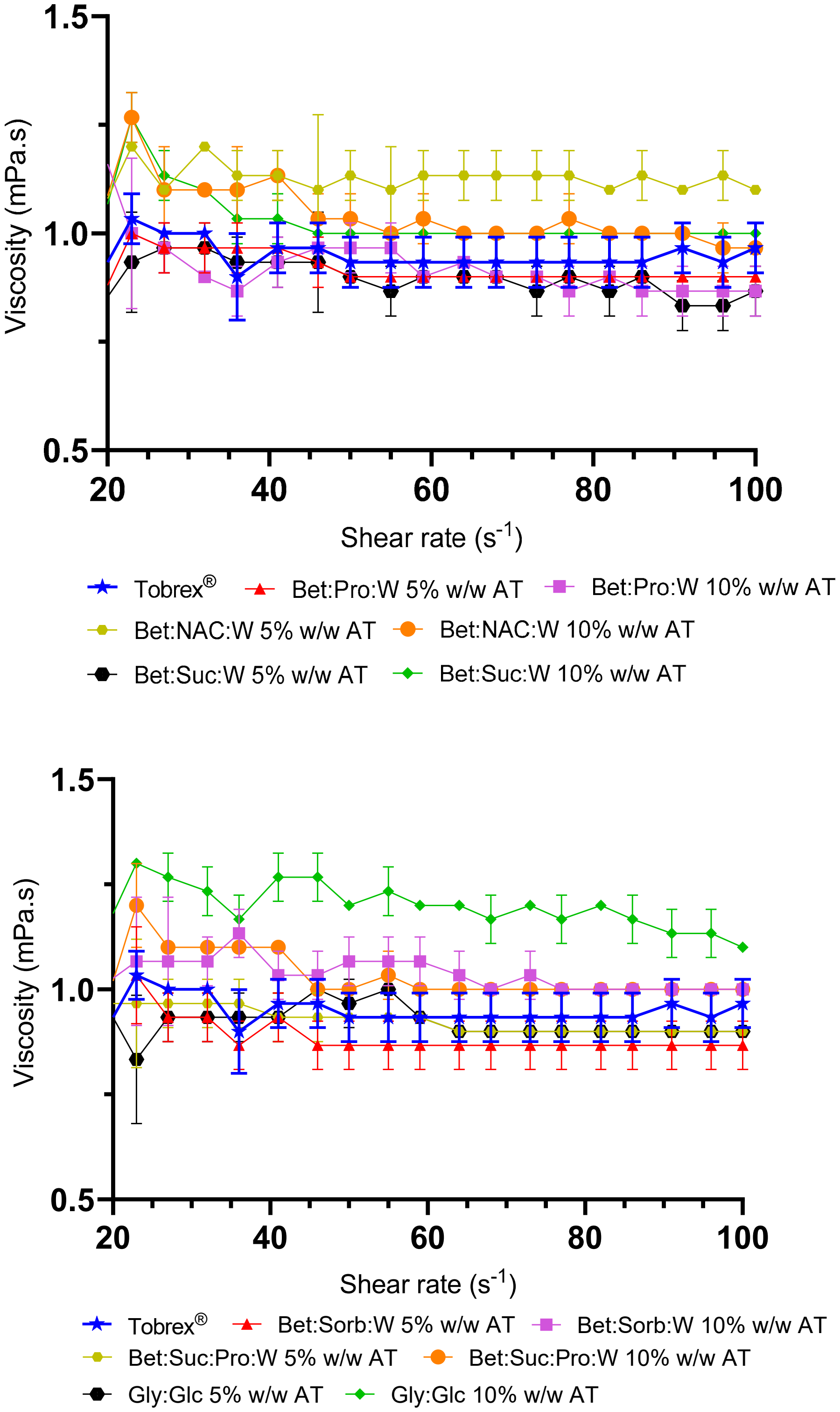

Figure S1. Viscosity as function of shear rate of NADES solutions and Tobrex®.

S2 Viscosity as a function of temperature

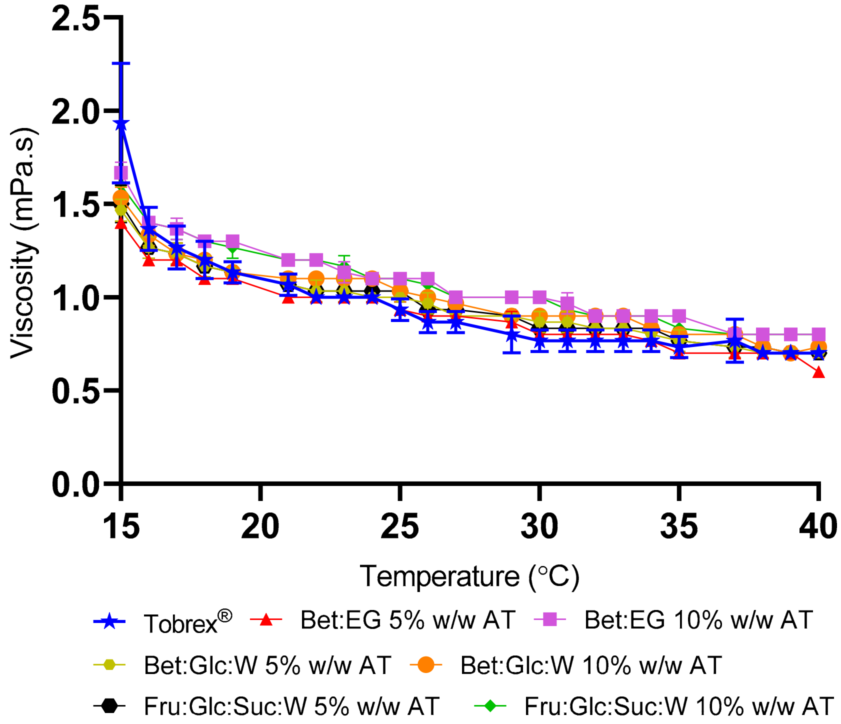

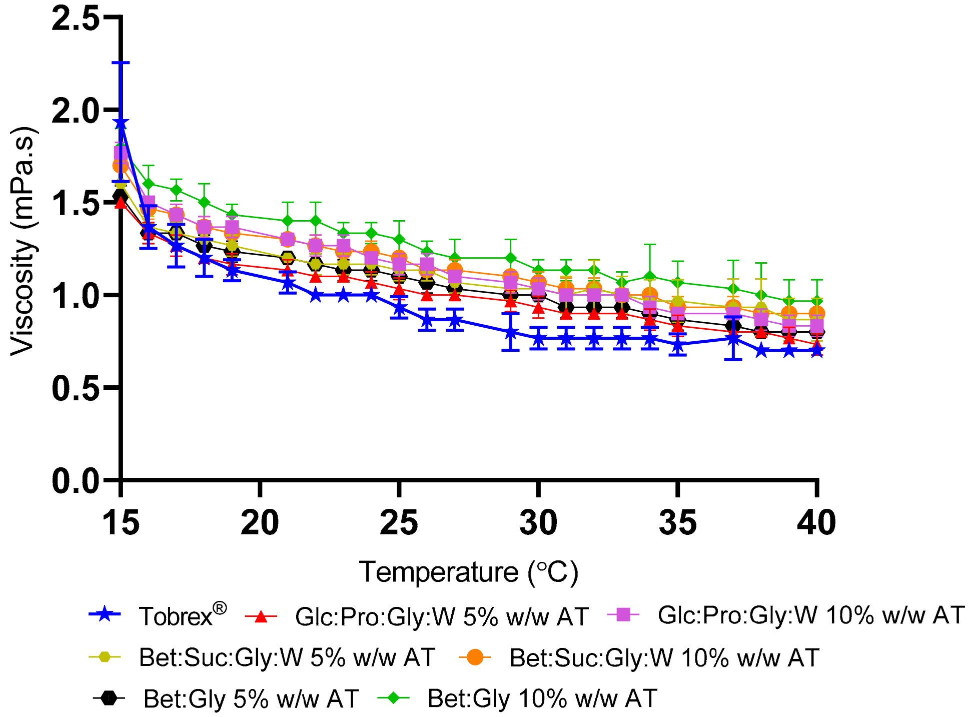

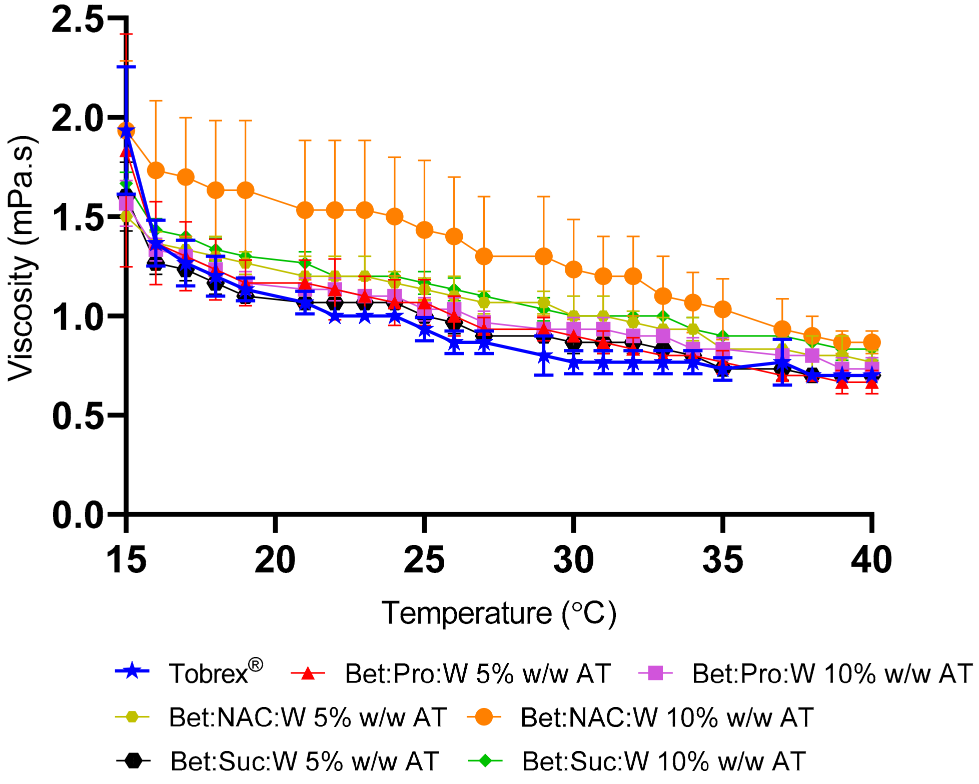

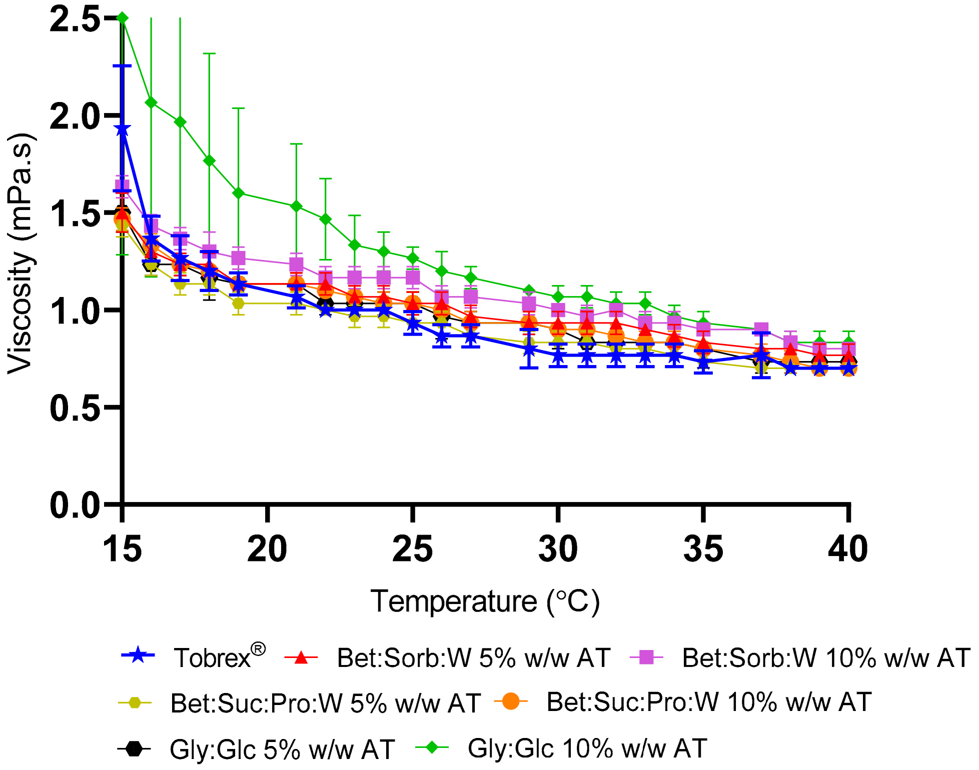

Figure S2. Viscosity *vs* temperature of NADES solutions and Tobrex®.

Table S1. Experimental viscosity data of Tobrex®, HPMC and NADES solutions(5% and 10%, w/v).

Temperature (ºC)	Tobex®		Bet:EG 5% w/w AT		Bet:EG 10% w/w AT		Bet:Glc:W 5% w/w AT		Bet:Glc:W 10% w/w AT	
	Viscosity (mPa)	SD	Viscosity (mPa)	SD	Viscosity (mPa)	SD	Viscosity (mPa)	SD	Viscosity (mPa)	SD
15.000	1.933	0.321	1.400	0.000	1.667	0.058	1.467	0.058	1.533	0.058
16.000	1.367	0.115	1.200	0.000	1.400	0.000	1.267	0.058	1.333	0.058
17.000	1.267	0.115	1.200	0.000	1.367	0.058	1.233	0.058	1.233	0.058
18.000	1.200	0.100	1.100	0.000	1.300	0.000	1.167	0.058	1.200	0.000
19.000	1.133	0.058	1.100	0.000	1.300	0.000	1.133	0.058	1.133	0.058
21.000	1.067	0.058	1.000	0.000	1.200	0.000	1.067	0.058	1.100	0.000
22.000	1.000	0.000	1.000	0.000	1.200	0.000	1.033	0.058	1.100	0.000
23.000	1.000	0.000	1.000	0.000	1.133	0.058	1.033	0.058	1.100	0.000
24.000	1.000	0.000	1.000	0.000	1.100	0.000	1.000	0.000	1.100	0.000
25.000	0.933	0.058	0.933	0.058	1.100	0.000	1.000	0.000	1.033	0.058
26.000	0.867	0.058	0.900	0.000	1.100	0.000	0.967	0.058	1.000	0.000
27.000	0.867	0.058	0.900	0.000	1.000	0.000	0.900	0.000	0.967	0.058
29.000	0.800	0.100	0.867	0.058	1.000	0.000	0.900	0.000	0.900	0.000
30.000	0.767	0.058	0.800	0.000	1.000	0.000	0.867	0.058	0.900	0.000
31.000	0.767	0.058	0.800	0.000	0.967	0.058	0.867	0.058	0.900	0.000
32.000	0.767	0.058	0.800	0.000	0.900	0.000	0.833	0.058	0.900	0.000
33.000	0.767	0.058	0.800	0.000	0.900	0.000	0.833	0.058	0.900	0.000
34.000	0.767	0.058	0.767	0.058	0.900	0.000	0.800	0.000	0.833	0.058
35.000	0.733	0.058	0.700	0.000	0.900	0.000	0.767	0.058	0.800	0.000
37.000	0.767	0.115	0.700	0.000	0.800	0.000	0.733	0.058	0.800	0.000
38.000	0.700	0.000	0.700	0.000	0.800	0.000	0.700	0.000	0.733	0.058
39.000	0.700	0.000	0.700	0.000	0.800	0.000	0.700	0.000	0.700	0.000
40.000	0.700	0.000	0.600	0.000	0.800	0.000	0.700	0.000	0.733	0.058

Table S1. Experimental viscosity data of Tobrex®, HPMC and NADES solutions(5% and 10%, w/v) (cont.)

Temperature (ºC)	Fru:Glc:Suc:W 5% w/w AT		Fru:Glc:Suc:W 10% w/w AT		Glc:Pro:Gly:W 5% w/w AT		Glc:Pro:Gly:W 10% w/w AT	
	Viscosity (mPa)	SD	Viscosity (mPa)	SD	Viscosity (mPa)	SD	Viscosity (mPa)	SD
15.000	1.500	0.100	1.600	0.100	1.500	0.000	1.767	0.058
16.000	1.267	0.058	1.400	0.000	1.333	0.058	1.500	0.000
17.000	1.233	0.058	1.367	0.058	1.267	0.058	1.433	0.058
18.000	1.167	0.058	1.300	0.000	1.200	0.000	1.367	0.058
19.000	1.133	0.058	1.267	0.058	1.167	0.058	1.367	0.058
21.000	1.067	0.058	1.200	0.000	1.133	0.058	1.300	0.000
22.000	1.033	0.058	1.200	0.000	1.100	0.000	1.267	0.058
23.000	1.033	0.058	1.167	0.058	1.100	0.000	1.267	0.058
24.000	1.033	0.058	1.100	0.000	1.067	0.058	1.200	0.000
25.000	1.033	0.058	1.100	0.000	1.033	0.058	1.167	0.058
26.000	0.933	0.058	1.067	0.058	1.000	0.000	1.167	0.058
27.000	0.933	0.058	1.000	0.000	1.000	0.000	1.100	0.000
29.000	0.900	0.000	1.000	0.000	0.967	0.058	1.067	0.058
30.000	0.833	0.058	1.000	0.000	0.933	0.058	1.033	0.058
31.000	0.833	0.058	0.933	0.058	0.900	0.000	1.000	0.000
32.000	0.833	0.058	0.900	0.000	0.900	0.000	1.000	0.000
33.000	0.833	0.058	0.900	0.000	0.900	0.000	1.000	0.000
34.000	0.833	0.058	0.900	0.000	0.867	0.058	0.933	0.058
35.000	0.767	0.058	0.833	0.058	0.833	0.058	0.900	0.000
37.000	0.733	0.058	0.800	0.000	0.800	0.000	0.900	0.000
38.000	0.733	0.058	0.800	0.000	0.800	0.000	0.867	0.058
39.000	0.700	0.000	0.800	0.000	0.767	0.058	0.833	0.058
40.000	0.700	0.000	0.800	0.000	0.733	0.058	0.833	0.058

Table S1. Experimental viscosity data of Tobrex®, HPMC and NADES solutions(5% and 10%, w/v) (cont.)

Temperature (ºC)	Bet:Suc:Gly:W 5% w/w AT		Bet:Suc:Gly:W 10% w/w AT		Bet:Gly 5% w/w AT		Bet:Gly 10% w/w AT	
	Viscosity (mPa)	SD	Viscosity (mPa)	SD	Viscosity (mPa)	SD	Viscosity (mPa)	SD
15.000	1.600	0.000	1.700	0.100	1.533	0.058	1.800	0.100
16.000	1.367	0.058	1.467	0.058	1.333	0.058	1.600	0.100
17.000	1.333	0.058	1.433	0.058	1.333	0.058	1.567	0.058
18.000	1.300	0.000	1.367	0.058	1.267	0.058	1.500	0.100
19.000	1.267	0.058	1.333	0.058	1.233	0.058	1.433	0.058
21.000	1.200	0.000	1.300	0.000	1.200	0.000	1.400	0.100
22.000	1.167	0.058	1.267	0.058	1.167	0.058	1.400	0.100
23.000	1.167	0.058	1.233	0.058	1.133	0.058	1.333	0.058
24.000	1.167	0.058	1.233	0.058	1.133	0.058	1.333	0.058
25.000	1.133	0.058	1.200	0.000	1.100	0.000	1.300	0.100
26.000	1.133	0.058	1.133	0.058	1.067	0.058	1.233	0.058
27.000	1.067	0.058	1.133	0.058	1.033	0.058	1.200	0.100
29.000	1.033	0.058	1.100	0.000	1.000	0.000	1.200	0.100
30.000	1.033	0.058	1.067	0.058	1.000	0.000	1.133	0.058
31.000	1.000	0.100	1.033	0.058	0.933	0.058	1.133	0.058
32.000	1.033	0.153	1.033	0.058	0.933	0.058	1.133	0.058
33.000	1.000	0.100	1.000	0.000	0.933	0.058	1.067	0.058
34.000	0.967	0.115	1.000	0.000	0.900	0.000	1.100	0.173
35.000	0.967	0.115	0.933	0.058	0.867	0.058	1.067	0.115
37.000	0.933	0.153	0.933	0.058	0.833	0.058	1.033	0.153
38.000	0.933	0.153	0.900	0.000	0.800	0.000	1.000	0.173
39.000	0.867	0.115	0.900	0.000	0.800	0.000	0.967	0.115
40.000	0.867	0.115	0.900	0.000	0.800	0.000	0.967	0.115

Table S1. Experimental viscosity data of Tobrex®, HPMC and NADES solutions(5% and 10%, w/v) (cont.)

Temperature (ºC)	Bet:Pro:W 5% w/w AT		Bet:Pro:W 10% w/w AT		Bet:NAC:W 5% w/w AT		Bet:NAC:W 10% w/w AT	
	Viscosity (mPa)	SD	Viscosity (mPa)	SD	Viscosity (mPa)	SD	Viscosity (mPa)	SD
15.000	1.833	0.586	1.567	0.115	1.500	0.000	1.933	0.351
16.000	1.367	0.208	1.333	0.058	1.367	0.058	1.733	0.351
17.000	1.300	0.173	1.300	0.000	1.333	0.058	1.700	0.300
18.000	1.233	0.153	1.233	0.058	1.300	0.100	1.633	0.351
19.000	1.167	0.115	1.167	0.058	1.267	0.058	1.633	0.351
21.000	1.167	0.115	1.133	0.058	1.200	0.100	1.533	0.351
22.000	1.133	0.153	1.133	0.058	1.200	0.100	1.533	0.351
23.000	1.100	0.100	1.100	0.000	1.200	0.100	1.533	0.351
24.000	1.067	0.115	1.100	0.000	1.167	0.058	1.500	0.300
25.000	1.067	0.115	1.033	0.058	1.133	0.058	1.433	0.351
26.000	1.000	0.100	1.033	0.058	1.100	0.100	1.400	0.300
27.000	0.933	0.058	0.967	0.058	1.067	0.058	1.300	0.300
29.000	0.933	0.058	0.933	0.058	1.067	0.058	1.300	0.300
30.000	0.900	0.000	0.933	0.058	1.000	0.100	1.233	0.252
31.000	0.867	0.058	0.933	0.058	1.000	0.100	1.200	0.200
32.000	0.833	0.058	0.900	0.000	0.967	0.058	1.200	0.200
33.000	0.800	0.000	0.900	0.000	0.933	0.058	1.100	0.200
34.000	0.800	0.000	0.833	0.058	0.933	0.058	1.067	0.153
35.000	0.767	0.058	0.833	0.058	0.833	0.058	1.033	0.153
37.000	0.700	0.000	0.800	0.000	0.833	0.058	0.933	0.153
38.000	0.700	0.000	0.800	0.000	0.800	0.100	0.900	0.100
39.000	0.667	0.058	0.733	0.058	0.800	0.100	0.867	0.058
40.000	0.667	0.058	0.733	0.058	0.767	0.058	0.867	0.058

Table S1. Experimental viscosity data of Tobrex®, HPMC and NADES solutions(5% and 10%, w/v) (cont.)

Temperature (ºC)	Bet:Suc:W 5% w/w AT		Bet:Suc:W 10% w/w AT		Bet:Sorb:W 5% w/w AT		Bet:Sorb:W 10% w/w AT	
	Viscosity (mPa)	SD	Viscosity (mPa)	SD	Viscosity (mPa)	SD	Viscosity (mPa)	SD
15.000	1.600	0.173	1.667	0.058	1.500	0.100	1.633	0.058
16.000	1.267	0.058	1.433	0.058	1.300	0.000	1.433	0.058
17.000	1.233	0.058	1.400	0.000	1.233	0.058	1.367	0.058
18.000	1.167	0.058	1.333	0.058	1.233	0.058	1.300	0.100
19.000	1.100	0.000	1.300	0.000	1.133	0.058	1.267	0.058
21.000	1.067	0.058	1.267	0.058	1.133	0.058	1.233	0.058
22.000	1.067	0.058	1.200	0.000	1.133	0.058	1.167	0.058
23.000	1.067	0.058	1.200	0.000	1.067	0.058	1.167	0.058
24.000	1.067	0.058	1.200	0.000	1.067	0.058	1.167	0.058
25.000	1.000	0.000	1.167	0.058	1.033	0.058	1.167	0.058
26.000	0.967	0.058	1.133	0.058	1.033	0.058	1.067	0.058
27.000	0.900	0.000	1.100	0.000	0.967	0.058	1.067	0.058
29.000	0.900	0.000	1.033	0.058	0.933	0.058	1.033	0.058
30.000	0.867	0.058	1.000	0.000	0.933	0.058	1.000	0.000
31.000	0.867	0.058	1.000	0.000	0.933	0.058	0.967	0.058
32.000	0.867	0.058	1.000	0.000	0.933	0.058	1.000	0.000
33.000	0.833	0.058	1.000	0.000	0.900	0.000	0.933	0.058
34.000	0.800	0.000	0.933	0.058	0.867	0.058	0.933	0.058
35.000	0.733	0.058	0.900	0.000	0.833	0.058	0.900	0.000
37.000	0.733	0.058	0.900	0.000	0.800	0.000	0.900	0.000
38.000	0.700	0.000	0.867	0.058	0.800	0.000	0.833	0.058
39.000	0.700	0.000	0.833	0.058	0.767	0.058	0.800	0.000
40.000	0.700	0.000	0.833	0.058	0.767	0.058	0.800	0.000

Table S1. Experimental viscosity data of Tobrex®, HPMC and NADES solutions(5% and 10%, w/v) (cont.)

Temperature (ºC)	Bet:Suc:Pro:W 5% w/w AT		Bet:Suc:Pro:W 10% w/w AT		Gly:Glc 5% w/w AT		Gly:Glc 10% w/w AT		Hypromellose 0,3% PBS	
	Viscosity (mPa)	SD	Viscosity (mPa)	SD	Viscosity (mPa)	SD	Viscosity (mPa)	SD	Viscosity (mPa)	SD
15.000	1.433	0.058	1.467	0.058	1.500	0.100	2.500	1.217	2.900	0.173
16.000	1.233	0.058	1.333	0.058	1.233	0.058	2.067	0.896	2.667	0.115
17.000	1.133	0.058	1.233	0.058	1.233	0.058	1.967	0.723	2.567	0.115
18.000	1.133	0.058	1.200	0.000	1.167	0.115	1.767	0.551	2.433	0.153
19.000	1.033	0.058	1.133	0.058	1.133	0.058	1.600	0.436	2.333	0.153
21.000	1.033	0.058	1.133	0.058	1.133	0.058	1.533	0.321	2.267	0.115
22.000	1.000	0.000	1.100	0.000	1.033	0.058	1.467	0.208	2.167	0.115
23.000	0.967	0.058	1.067	0.058	1.033	0.058	1.333	0.153	2.167	0.115
24.000	0.967	0.058	1.033	0.058	1.033	0.058	1.300	0.100	2.067	0.115
25.000	0.933	0.058	1.033	0.058	1.033	0.058	1.267	0.058	2.000	0.100
26.000	0.933	0.058	1.000	0.000	0.967	0.115	1.200	0.100	1.933	0.058
27.000	0.867	0.058	0.933	0.058	0.933	0.058	1.167	0.058	1.867	0.115
29.000	0.833	0.058	0.933	0.058	0.933	0.058	1.100	0.000	1.800	0.100
30.000	0.833	0.058	0.900	0.000	0.900	0.100	1.067	0.058	1.733	0.058
31.000	0.833	0.058	0.900	0.000	0.833	0.058	1.067	0.058	1.667	0.058
32.000	0.800	0.000	0.867	0.058	0.833	0.058	1.033	0.058	1.667	0.058
33.000	0.800	0.000	0.833	0.058	0.833	0.058	1.033	0.058	1.633	0.058
34.000	0.767	0.058	0.833	0.058	0.833	0.058	0.967	0.058	1.567	0.115
35.000	0.733	0.058	0.800	0.000	0.800	0.100	0.933	0.058	1.533	0.058
37.000	0.700	0.000	0.767	0.058	0.733	0.058	0.900	0.000	1.433	0.058
38.000	0.700	0.000	0.733	0.058	0.733	0.058	0.833	0.058	1.433	0.058
39.000	0.700	0.000	0.700	0.000	0.733	0.058	0.833	0.058	1.433	0.058
40.000	0.700	0.000	0.700	0.000	0.733	0.058	0.833	0.058	1.433	0.153

S3. Solubility studies

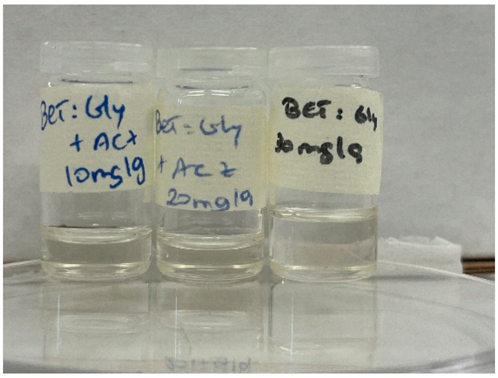

Figure S3. Example of ACZ dissolved in NADES.

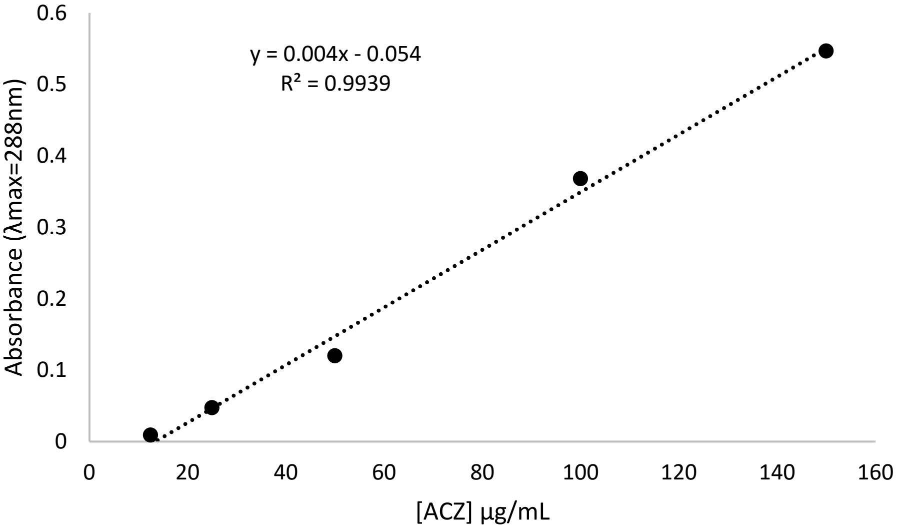

Figure S4. Calibration Curve of Acetalozamide.

Table S2. Quantification of ACZ in 5% aqueous solutions of NADES/ACZ mixtures.

[ACZ] in NADES (mg/g)	$[ACZ]_{teo}$ in 5% aq NADES+ACZ (mg/mL)	[ACZ]exp in 5% aq. NADES+ACZ (mg/mL)								
		Bet:Eg	Bet:Glc	Glc:Pro:Gly	Bet:Gly:Suc	Bet:Gly	Bet:NAC	Bet:Suc	Bet:Suc:Pro	Gly:Glc
**10**	0.5	0.57±0.04	0.57±0.11	0.56±0.08	0.48±0.1	0.49±0.06	0.55±0.04	0.57±0.1	0.51±0.04	0.47±0.04
**20**	1	1.10±0.1	1.16±0.02	1.14±0.05	1.05±0.12	1.01±0.04	1.12±0.07	1.00±0.13	0.94±0.12	-
**30**	1.5	1.51±0.01	1.58±0.04	-	-	1.41±0.07	1.53±0.06	1.31±0.17	-	-
